# The Feasibility of the Three-Dimensional Footswitch-Operated Robotic Arm Exoscope for Cochlear Implant Surgery

**DOI:** 10.1097/MAO.0000000000003952

**Published:** 2023-07-18

**Authors:** Eerika Karinen, Matti Iso-Mustajärvi, Aarno Dietz

**Affiliations:** ∗ Department of Otorhinolaryngology, Satasairaala Hospital, Pori, Finland; † Department of Otorhinolaryngology, Kuopio University Hospital, Kuopio, Finland; ‡ Institute of Clinical Medicine, Otorhinolaryngology, University of Eastern Finland, Kuopio, Finland

**Keywords:** Cochlear implantation, Complications, Ergonomics, Exoscope, Image quality, Surgical outcomes, Three-dimensional

## Abstract

**Objective:**

To compare the three-dimensional (3D) footswitch-operated robotic arm exoscope with the operating microscope (OM) in cochlear implant surgery.

**Study design:**

Matched case–control study.

**Patients:**

Cochlear implantation was performed with the exoscope on unselected patients with normal temporal anatomy. The control group that underwent cochlear implantation with the OM was case matched with respect to age, anatomy, surgical technique, and type of anesthesia.

**Interventions:**

Cochlear implantation performed with the 3D exoscope.

**Main Outcome measures:**

Surgical time, occupation of the operation theater, surgical results, and user experience evaluated by a questionnaire.

**Results:**

Eleven patients (13 ears) were successfully operated on with the exoscope. In the exoscope group, we observed one minor intraoperative complication, where the middle dura was exposed during mastoidectomy. Although no clear preference was evident for either device in the overall rating, the subdomain rating revealed that the exoscope’s image quality was deemed inferior, especially at higher magnifications where pixelation became noticeable. The exoscope received higher scores for usability, particularly excelling in terms of surgeon’s ergonomic and comfortability. There was a statistically significant difference in mean surgical time, 146 and 129 min for the exoscope and OM group, respectively.

**Conclusions:**

Cochlear implant surgery was found to be feasible with a 3D exoscope. However, there is a learning curve to overcome regarding handling and the different quality of the image. The exoscope provides better ergonomics for the surgeon.

## INTRODUCTION

Since 1951, the operating microscope (OM) was used in both oto- and neurosurgery (1). Subsequently, the OM became the gold standard for otosurgery because of its ability to illuminate and magnify the surgical field. Limitations encountered with current OMs are mainly related to poor ergonomics due to the fixed optical axis. In fact, the use of OMs has been identified as a risk factor for work-related musculoskeletal disorders in surgeons (2). Additionally, static posture during surgery with the OM may cause muscle fatigue and can lead to suboptimal task performance (3).

Three-dimensional (3D) exoscopes have been developed to overcome these limitations. The most recent exoscopes comprise of two 2k digital cameras with optical zoom and deliver a detailed 3D image of the surgical field via 3D monitors observed with 3D glasses (4). Currently exoscopes are used in neurosurgical procedures as these are mostly open corridor surgeries (4–6). For surgery in deep recesses (e.g., narrow posterior tympanotomy), most of the light remain outside the surgical corridor, which may deteriorate image quality (7), limiting the application of the exoscope to line of sight approaches, identical to the traditional OM. The literature about exoscope in otologic surgery is still sparse and therefore warrants additional studies to establish the role of current devices in otology (6–11). The differences between exoscopes and OMs concerning handling, image quality, surgical view, depth of focus, ergonomics, as well as observer experiences and education with cochlear implant (CI) surgery have yet to be determined (7–9,12,13).

The study’s aim was to compare the exoscope with the OM in CI surgery. We investigated following outcomes: (i) surgical results, (ii) operation time and overall occupation time in the OR, and (iii) surgeon’s opinion assessed by a questionnaire.

## MATERIALS AND METHODS

**FIG. 1:**
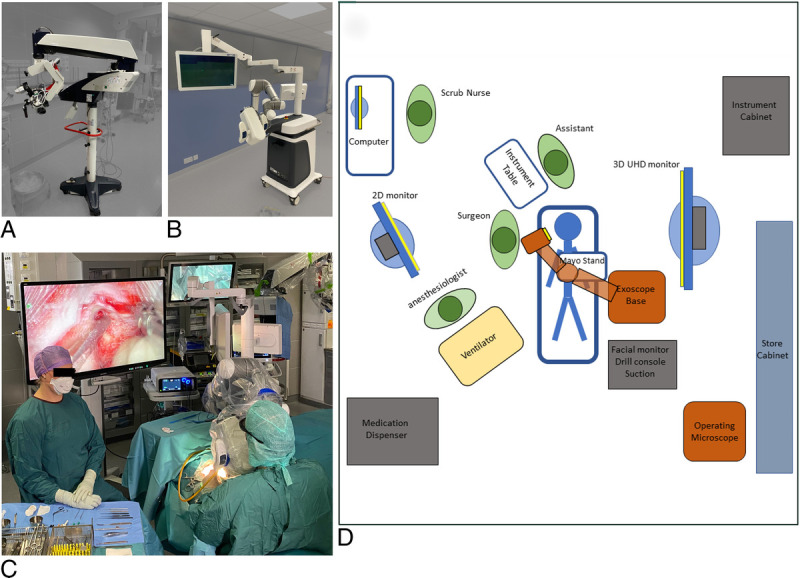
*A*, The operating microscope; *B*, the footswitch-operated robotic arm exoscope; *C*, the exoscope in action during cochlear implant surgery; *D*, operation room setup with device footprints of the exoscope and 3D monitor screen.

This is a retrospective case–control trial (institutional approval no. 5551886) from the Department of Otorhinolaryngology of Kuopio University Hospital. Two authors (M.I. and A.D.) performed surgeries in 11 consecutive patients with a footswitch-operated robotic arm exoscope (Aesculap Aeos, Braun, Kronberg im Taunus, Germany) between November 2021 and March 2022. The camera head was positioned in front of the surgeon, and a 55-inch 4K 3D monitor was positioned at around 1.5 to 2 m of the surgeon (Fig. 1B–D). A retrospective control group operated on with an OM (Leica F40 OM, Wetzlar, Germany) between February 2019 and March 2022 was matched to the exoscope group regarding age, anatomy, surgical technique, and the type of anesthesia. At our institution, surgery under local anesthesia (LA) is preferred for elderly patients (>75 years) if significant comorbidities are present (14). All patients had normal temporal bone anatomy and fulfilled criteria for cochlear implantation. Preoperative high-resolution computed tomography (CT) and postoperative cone beam CT were performed in adult patients. Patient demographics are presented in Table 1.

**TABLE 1:** Patient demographics, surgical data, and comparison of the groups

Exoscope Group									
Ear	Side	Age (yr)	Sex	Etiology	Anesthesia	Operation Time (min)	OR Occupation Time (min)	Complication	Surgeon
Exoscope group									
1*	Right	1	M	NSSNHL	General	105	238	No	1
2*	Left	1	M	NSSNHL	General	111	170	No	1
3*	Right	3	M	NSSNHL	General	132	234	No	2
4*	Left	3	M	NSSNHL	General	190	233	No	2
5	Left	16	M	SNHL	General	139	193	No	1
6	Right	33	F	SSNHL	General	115	198	No	1
7	Right	41	M	SNHL	Local	155	211	No	1
8	Right	47	F	SSD	General	119	188	No	1
9	Right	56	F	SNHL	General	199	273	No	2
10	Left	70	M	Otosclerosis	General	143	251	Dural exposure	1
11	Right	70	M	ARHL	General	168	263	No	2
12	Left	75	F	MD	General	180	263	No	1
13	Left	75	M	ARHL	Local	146	211	No	2
Mean		38				146	225		
Median		41				143	233		
Operating microscope group									
1*	Right	1	F	NASNHL	General	159	205	No	1
2*	Left	1	F	NASNHL	General	163	209	No	1
3*	Right	1	M	NSSNHL	General	164	382	No	1
4*	Left	1	M	NSSNHL	General	185	403	No	2
5	Right	9	M	NSSNHL	General	118	192	No	1
6	Right	29	F	SNHL	General	112	177	No	2
7	Right	41	M	SNHL	General	126	206	No	1
8	Left	46	M	SNHL	General	103	194	No	1
9	Left	52	M	SSD	Local	98	163	No	1
10	Left	55	F	Otosclerosis	General	92	180	No	1
11	Left	67	F	ARHL	General	131	197	No	2
12	Right	74	F	Otosclerosis	Local	99	154	No	1
13	Right	78	M	ARHL	General	122	185	No	1
Mean		35				129	219		
Median		41				122	194		
Comparison between groups									
*p*	1.000	0.972	0.480	0.357	1.000	0.173	0.422	0.317	0.414

M, male; F, female; SNHL, sensorineural hearing loss of unknown etiology; MD, Ménière’s disease; SSNHL, syndromic sensorineural hearing loss; NSSNHL, nonsyndromic sensorineural hearing loss; SSD, single-sided deafness; ARHL, age-related sensorineural hearing loss; NASNHL, neonatal acquired sensorineural hearing loss; OR, operating room.

The participating surgeons filled in a questionnaire to systematically assess their opinion and experience with the exoscope (surgeon 1 = 8 ears; surgeon 2 = 5 ears). The answers were based on a subjective assessment of previous recollection of the same surgical procedure performed with OM. The operation experience was evaluated with the following scoring system: 0 = no preference, +1 = exoscope is slightly preferred, +2 exoscope is strongly preferred, −1 OM is preferred, and −2 OM is strongly preferred. Additionally, we collected data of the operative time and the overall occupation time in the OR.

The Mann–Whitney *U* test was used for statistical analysis and group comparison. SPSS 27.0 software (IBM SPSS, Armonk, NY) was used.

## RESULTS

All 11 patients (13 ears) were successfully implanted with the exoscope. Accordingly, 11 patients (13 ears) were enrolled for the control group. There were no statistically significant differences in sex, age, and etiology or modality of anesthesia between the groups.

There was no need for conversion from the exoscope to the OM in any case. All electrode insertions were performed as preoperatively planned with adequate placement in every case. One minor complication occurred in the exoscope group, where a small area (Ø ~ 4 mm) of the middle fossa dura was exposed during mastoidectomy. The defect was successfully reconstructed with bone paté and fibrin glue (Table 1). No complications were recorded in the control group.

According to the questionnaire, overall image quality and image resolution was deemed inferior to the OM by both surgeons as was the depth perception. However, magnification (i.e., optical zoom), lighting of the surgical field, and also through narrow corridors (e.g., round window visualization) were found equally good or even slightly better compared with the OM. Both surgeons evaluated ergonomics and comfortability afforded by the exoscope to be superior compared with the OM. Additionally, in surgeries with the exoscope, the patient’s head could be positioned more comfortably without need for undue extension. This was found especially beneficial for patients operated under LA. Regarding the performance of the subtasks related to CI surgery, the surgeons expressed no clear preference for either the exoscope or OM. However, for challenging cases, the OM was preferred due its superior image quality. The mean score for surgeon 1 was 0.3, indicating a slight preference toward the exoscope, and it was 0.0 for surgeon 2, indicating no clear preference for either device (Table 2).

**TABLE 2:** The surgeons rating for each device

	Surgeon 1	Surgeon 2
Usability (mean)	0.8	0.6
Ergonomy	2	2
Comfortability	2	1
The positioning of the patient	2	1
Positioning of the exoscope in the operating room	−1	0
Need for re-adjustments	1	0
Assimilation to the use of the instrument	0	−1
The use of the instrument	0	0
The steering response of the instrument	0	1
The acclimation of the instrument to positional changes	1	1
Visualization (mean)	−0.8	0
Differentiation of tissue	−1	−1
Recognition of anatomical structures	−1	−1
Quality of the image	−2	−1
Stability of the image	0	0
Depth perception	−1	−1
Lighting of the surgical view	0	2
Lighting in small surgical corridors	0	2
Lighting in the operating room	−1	0
Performing cochlear implantation (mean)	−0.3	0.3
Skin incision and temporal flap	0	0
Subtotal mastoidectomy	0	0
Posterior tympanotomy	0	−1
Thinning of the posterior ear canal	−1	0
Drilling of the implant bed	1	1
Drilling of the bony overhang of the round window	−1	1
Performing the cochlear implant electrode insertion	−1	1
Overall mean	0.0	0.3
Number of previous cadaveric training sessions	2	5
Preference	No preference	Exoscope

Scoring: 0 = no preference, +1 = exoscope is slightly preferred, +2 exoscope is strongly preferred, −1 OM is preferred, −2 OM is strongly preferred.

The mean overall occupation time of the OR was 225 and 219 min for the exoscope group and the OM group, respectively. The mean time for the actual procedure was 146 min (SD 31) for the exoscope group and 129 min (SD 30) for the OM group; this difference was statistically significant (Table 1).

## DISCUSSION

To the best of our knowledge, this is the first case–control study investigating the feasibility of using a footswitch robotic arm 3D exoscope in CI surgery. In general, the exoscope proved its usefulness as all procedures were successfully performed and with comparable outcomes as would have been achieved with the OM. This finding is in line with previous studies investigating the use of the exoscope, although these have been mainly restricted to neurosurgical procedures (5,8–10,12,15).

The main disadvantage of the exoscope is related to the lower quality of image, which can make the differentiation of anatomic structures and different tissues more difficult for learners in comparison to the OM. The unfamiliarity to the exoscope image was considered a contributing factor for the exposure of the middle fossa dura (Ear 10). Accordingly, both surgeons found that familiarization to the exoscopes’ image was required for a reliable identification of anatomical structures and different tissue qualities. However, with increasing experience, these structures and tissues could be easily and reliably identified. Thus, we found that there is a learning curve related not only to the handling of the exoscope but also to the comprehension of the different kind of image provided by the exoscope.

In their proof-of-concept study, Smith et al. (7) found that the main limitations of the exoscope are low light in small surgical corridors and pixelation at high magnification. In contrast to their finding, our study suggests that current exoscopes can perform rather well also in small surgical corridors such as visualization of the round window area through a narrow posterior tympanotomy. Although we did observe some pixelation at highest optical magnification, image quality remained adequate for every task of CI surgery (7).

**FIG. 2:**
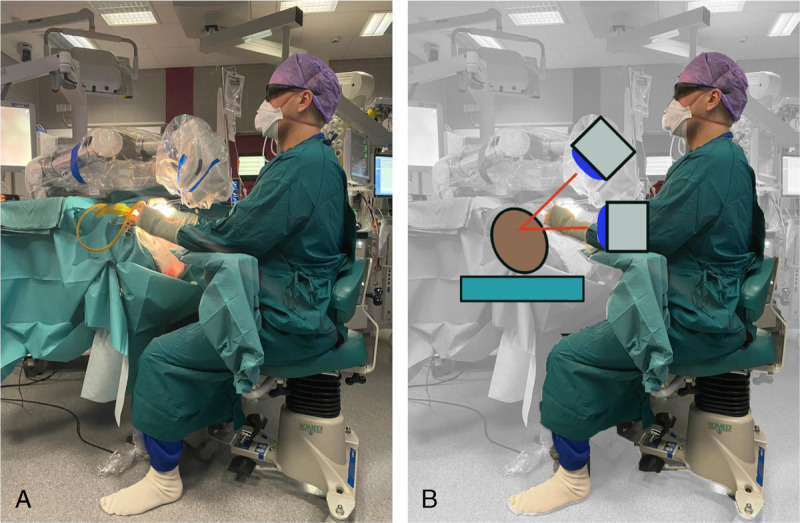
*A*, With the exoscope, the surgeon can adopt an ergonomic posture with the exoscope. *B*, The surgeon is not confined to an optical axis as with the operating microscope and he/she can adjust the camera head to different angles allowing for good ergonomics.

The main advantages of the exoscope are the significantly better ergonomics and quick adjustment of the 3D camera to achieve an optimal angle of view/exposure with little need for readjusting the OR table or the patient’s head. We found this especially beneficial for patients undergoing cochlear implantation under LA in whom a comfortable head position is essential to endure surgery. Likewise, tilting of the OR table is also restricted in awake patients. Because of these limitations, surgery under LA is ergonomically challenging for the surgeon. The exoscope significantly improves a surgeon’s posture, especially during surgeries performed under LA (Fig. 2).

The exoscope also enhances the opportunities for teaching. All members of the operating team, including residents, can follow the procedure while appreciating the same high-quality 3D image as the surgeon. It overcomes some of the challenges encountered when teaching residents/fellows as they will enjoy the same view of the surgery. We would argue that this represents a more efficient form of teaching (16).

The mean operation time was statistically significantly shorter for the OM, which is related to the long-standing routine with the OM. With repeated use of the exoscope, however, it can be expected that the surgeons will become more comfortable and more efficient.

The limitations of this study are related to the small sample size and to the novelty of the exoscope requiring training. During this study, the surgeons were still on their learning curve.

## CONCLUSION

This study established the feasibility of the 3D exoscope for CI surgery. Although the image quality using 2k cameras has not yet matched the level of the OM optics, the exoscope performed well in depth perception, magnification, and illumination in narrow surgical corridors. Familiarization is needed to operate the exoscope, but its adjustable viewing angle enhances surgeon ergonomics.
